# Effectiveness of bronchoscopy-assisted postoperative respiratory management in patients with lung cancer and impaired cough strength: a retrospective cohort study

**DOI:** 10.1097/MS9.0000000000003202

**Published:** 2025-03-28

**Authors:** Guofei Zhang, Lian Wang, Jia Han, Jiali Chen, Jiafeng Wu

**Affiliations:** a Department of Thoracic Surgery, the Second Affiliated Hospital of the Zhejiang University School of Medicine, Hangzhou, China; b Department of Thoracic Surgery, Hangzhou No. 9 People’s Hospital & Hangzhou Red Cross Hospital, Qiantang Branch, Hangzhou, China

**Keywords:** bronchoscopy-assisted postoperative respiratory management, cough strength, lung cancer, respiratory complications

## Abstract

**Background:**

Patients with lung cancer and impaired cough strength have an increased risk of postoperative respiratory complications. This study aimed to investigate the effectiveness of bronchoscopy-assisted postoperative respiratory management in reducing these complications.

**Materials and methods:**

This retrospective study included 781 lung cancer patients who received comprehensive postoperative respiratory management between April 2021 and May 2023 in a tertiary care setting. Cough strength was assessed on a scale of 0–5, and patients were categorized by secretion volume. Patients with a cough strength score ≤2 and moderate or higher secretions were identified for bronchoscopy-assisted management.

**Results:**

Twelve patients underwent bronchoscopy-assisted postoperative respiratory management. All 12 patients successfully recovered without requiring postoperative reintubation. Inflammatory marker levels significantly decreased after bronchoscopy and suctioning, with no in-hospital fatalities. The average postoperative hospital stay was 8.0 ± 5.5 days. Among the patients who did not require bronchoscopy-assisted suction, 71 experienced delayed discharge owing to various pulmonary complications, although none required reintubation.

**Conclusions:**

Bronchoscopy-assisted postoperative respiratory management was a promising strategy to prevent respiratory complications in patients with lung cancer and impaired cough strength. Our findings challenge the notion that weak airway competence is a contraindication for lung surgery. Early bronchoscopic intervention and diligent evaluation of airway secretions and cough strength offer substantial potential to improve patient outcomes.

**HIGHLIGHTS**

- This study demonstrates that bronchoscopy-assisted postoperative respiratory management effectively prevents respiratory complications in lung cancer patients with impaired cough strength, with all 12 patients successfully recovering without reintubation.
- A novel airway competence assessment model is introduced, integrating cough strength and secretion volume to guide clinical decision-making in high-risk surgical candidates.
- Early bronchoscopic intervention is shown to significantly improve postoperative outcomes, challenging the notion that weak airway competence should contraindicate lung surgery and providing new treatment pathways for vulnerable populations.

## Introduction

Respiratory complications following lung cancer surgery such as atelectasis, pneumonia, and acute respiratory distress syndrome continue to be significant concerns despite advancements in surgical techniques and perioperative care^[1-3]^. These complications pose a particularly serious threat to patients with compromised respiratory function stemming from various factors such as neurological or muscular disorders, comorbidities, and advanced age^[4,5]^.

Two pivotal factors emerge regarding the prediction of extubation outcomes after successful spontaneous breathing trials for ventilated patients: cough strength and endotracheal secretion volume^[6,7]^. A semiquantitative cough strength score ranging from 0 to 5, coupled with an endotracheal secretion assessment (categorized as none, mild, moderate, or abundant) are instrumental in predicting the likelihood of reintubation after planned extubation^[8]^. Notably, patients with a feeble cough (score ≤2) and those burdened by moderate-to-abundant secretions have an elevated risk of unsuccessful extubation^[6]^.

Bronchoscope-assisted sputum suction plays a crucial role in modulating the inflammatory response in patients with severe pneumonia^[9,10]^. Bronchoscopy-assisted postoperative respiratory management may be a promising strategy in response to the unique respiratory challenges faced by patients with lung cancer characterized by diminished cough strength and heightened secretion levels. However, previous studies indicate deficiencies in identifying patients who require postoperative bronchoscopy-assisted respiratory management. Traditional approaches may have inadequately accounted for individual variabilities, particularly in assessing postoperative cough strength and secretions, and addressing potential complications^[11,12]^. Therefore, we introduced the “airway competence assessment model” that evaluates cough strength and secretion levels to identify postoperative patients with lung cancer who would benefit from bronchoscopy-assisted respiratory management. This study aimed to share our experiences with this innovative approach and elucidate its efficacy in mitigating respiratory complications within this specific patient demographic. This cohort study has been reported in line with the STROCSS guidelines^[13]^.

## Materials and methods

### Patients

Our surgical team at the Second Affiliated Hospital of Zhejiang University School of Medicine performed surgeries on a consecutive cohort of 781 patients diagnosed with lung cancer from April 2021 to May 2023, all of whom underwent thorough postoperative respiratory management as an integral component of their care. Within this cohort, a subset of 12 patients who underwent bronchoscopy-assisted postoperative respiratory management was selected after a comprehensive assessment of their postoperative cough strength and endotracheal secretion extent.

The study adhered to the ethical principles outlined in the Declaration of Helsinki was approved by the institutional review board, and a waiver for informed consent was obtained owing to the retrospective nature of the study. The study was retrospectively registered in the Research Registry with the unique research registration number [researchregistry11029] (https://www.researchregistry.com/register-now#home/registrationdetails/67acb537bdc223176639fda9/).

### Postoperative airway competence assessments

Following extubation and transfer back to the ward after recovery in the postoperative care unit, patient airway competence (referring to the ability to generate a robust cough and expectorate endotracheal secretions) was meticulously evaluated to identify those requiring bronchoscopy-assisted postoperative respiratory management. This critical assessment was performed by a single observer (ZG) in collaboration with bedside nurses and respiratory therapists.

### Cough strength assessment

The primary criterion was voluntary cough strength. The observer employed a semi-objective scale ranged from 0 to 5 to effectively assess cough strength:

- 0: No cough when instructed
- 1: Visible movement, no audible cough
- 2: Weakly (barely) audible cough
- 3: Clearly audible cough
- 4: Stronger cough
- 5: Multiple sequential strong cough

### Secretion volume assessment

In addition to cough strength, the quantity of endotracheal secretions was meticulously evaluated by the same single observer. Patients were categorized into one of four groups based on a combination of personal observations and collective information from the nursing and respiratory therapy professionals. The secretion volume categories were defined as follows:

- No: Absence of endotracheal secretions
- Mild: Minimal endotracheal secretions
- Moderate: Moderate volume of endotracheal secretions
- Abundant: Significant volume of endotracheal secretions

### Identification of patients requiring bronchoscopy-assisted management

Patients with weak cough strength (grades 0–2) and moderate or high endotracheal secretion levels were identified as candidates for bronchoscopy-assisted postoperative respiratory management.

### Bronchoscopy-assisted postoperative respiratory management

A bronchoscopic procedure employing the BF-QT170 bronchoscope (Olympus Medical, Tokyo, Japan) was gently performed to minimize airway irritation. The nasal mucosa was lubricated and topically anesthetized using 2% lidocaine jelly. As the bronchoscope approached the vocal cords, lidocaine (2 mL per application) was sprayed through the bronchoscope channel to the level of the vocal cords and repeated three to four times. The bronchoscope was carefully inserted into the trachea, with simultaneous spraying of lidocaine to minimize discomfort after surface anesthesia of the vocal cords. It was crucial to avoid touching the tracheal wall with the bronchoscope tip as the bronchoscope advances to the carina.

Suctioning began on the side with fewer secretions before moving to the side with more secretions. If necessary, lidocaine spray was reapplied during this process until all secretions were effectively cleared from the airway. A sputum collection device was used to gather a portion of the sputum for bacterial culture and sensitivity testing, which guided antibiotic usage. The frequency and extent of suction were continually adjusted based on real-time assessments of secretion and cough efficacy.

### Surgical technique

The surgeries were conducted with patients in the lateral decubitus position under general anesthesia with double-lumen endotracheal intubation as previously detailed in our surgical technique descriptions^[14,15]^. Thoracoscopic surgery was performed using the single-port approach. The choice between lobectomy and sublobar resection was based on the lesion location and size, with simultaneous mediastinal lymph node dissection. Thoracic paravertebral block anesthesia with ropivacaine was administered via percutaneous puncture under direct visualization before closing the surgical incision.

### Follow-up and data collection

We initiated a follow-up protocol designed to assess the patient’s postoperative respiratory status and identify potential issues within 3 days of hospital discharge. The protocol involved a structured telephone call with the patients. Additionally, we scheduled a face-to-face follow-up visit within 2 weeks of discharge. During these visits, we assessed patients’ ongoing respiratory management needs, provided education on recognizing when to seek medical attention, and conducted routine chest computed tomography (CT) scans or radiographs to rule out pulmonary complications.

### Statistical analysis

Statistical analyses were performed using SPSS for Windows version 22 (IBM Corp., Armonk, NY, USA). Descriptive statistics were used to summarize the demographic and clinical characteristics of the study cohort. Continuous variables are presented as means with standard deviations, while categorical variables are expressed as counts and percentages.

## Results

**Figure 1.**
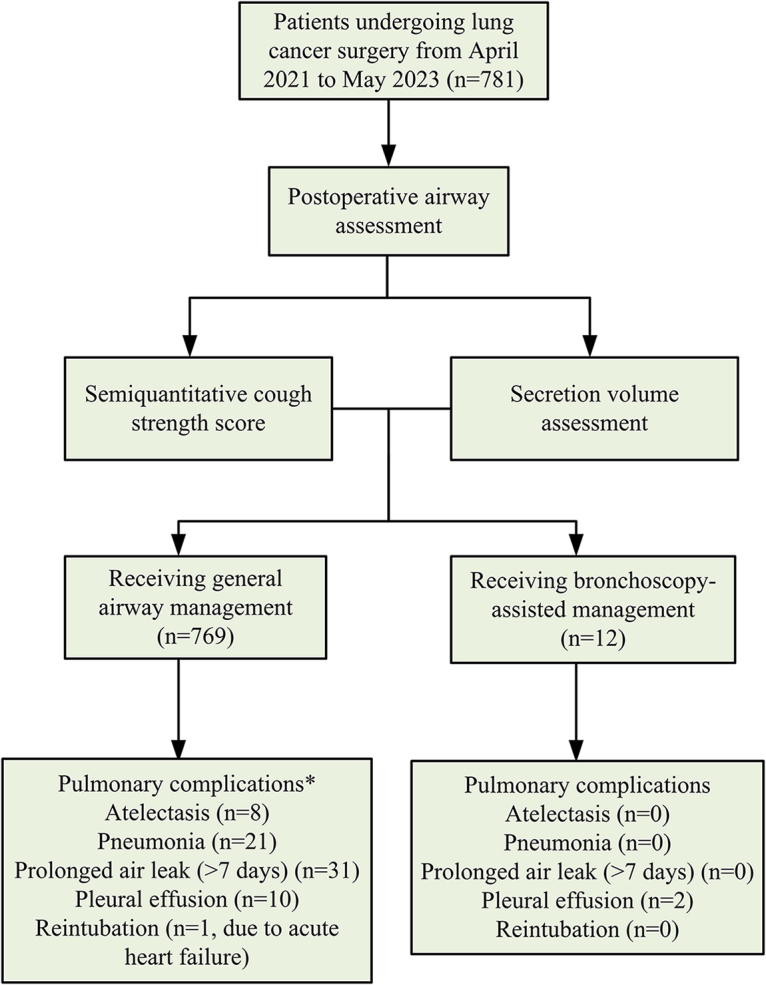
**Flowchart for patient inclusion and groups**.* reasons for delayed discharge owing to pulmonary complications (postoperative hospital stay > 5 days)

Among the 781 patients, 12 received bronchoscopy-assisted postoperative respiratory management based on a semiquantitative cough strength score ≤2 and moderate or greater magnitude of endotracheal secretions (Fig. 1). The age range of these patients spanned from 36 to 90 (mean age of 73.7 ± 13.4) years (Table 1). Notably, seven of these individuals had neurological conditions, one with post-polio sequelae and six with post-cerebral infarction sequelae, resulting in hemiplegia. Additionally, three patients were older and frail, while two had chronic bronchitis and emphysema.

**Table 1:** Characteristics and surgical details of patients receiving bronchoscopy-assisted postoperative respiratory management

ID	Sex	Age (years)	Pathology/tumor location	Comorbidities^a^	Surgery type^b^	Surgery duration (minutes)	Hospital stay (post-op stay)	Cough strength	Secretion volume	Bronchoscopy-assisted suctioning
1	M	75	SCC/RUL	Chronic bronchitis with emphysema^c^	Lobectomy	165	8 (6)	2	Abundant	1
2	M	67	SCC/RLL	Sequelae of cerebral infarction^c^	Lobectomy	225	15 (6)	1	Moderate	1
3	F	36	MEC/RML&RLL	Sequelae of poliomyelitis	Lobectomy	130	21 (7)	0	Abundant	3
4	F	72	AC/RUL	Sequelae of cerebral infarction	Sleeve resection	230	9 (8)	2	Abundant	2
5	M	70	LCNC/LUL	Sequelae of cerebral infarction	Lobectomy	140	24 (8)	1	Abundant	3
6	M	75	SC/LLL	Sequelae of cerebral infarction^d^	Segmentectomy	140	24 (8)	1	Abundant	2
7	M	90	SCC/LUL	Frailty	Segmentectomy	65	25 (9)	1	Abundant	1
8	M	85	SCC/RUL	Frailty	Segmentectomy	165	31 (25)	1	Abundant	1
9	M	78	SCC/LUL	Chronic bronchitis with emphysema	Lobectomy	120	9 (7)	1	Abundant	1
10	M	78	AC/LLL	Sequelae of cerebral infarction	Lobectomy	70	6 (4)	1	Abundant	1
11	M	79	SCC/LUL	Sequelae of cerebral Infarction^c^ post-esophagectomy	Lobectomy	220	22 (11)	1	Abundant	1
12	M	79	SCC/RLL	Frailty	Lobectomy	200	20 (14)	0	Abundant	1

AC, adenocarcinoma; F, female; M, male; MEC, mucinous epidermoid carcinoma; LCNC, large cell neuroendocrine carcinoma; LLL, left lower lobe; LUL, left upper lobe; RLL, right lower lobe; RML right middle lobe; RUL, right upper lobe; SC, sarcomatoid carcinoma; SCC, squamous cell carcinoma.

^a^ Main comorbidities leading to reduced cough strength.

^b^ All surgeries were performed under video-assisted thoracoscopic surgery.

^c^ Received preoperative neoadjuvant chemotherapy and immunotherapy.

^d^ Reoperation owing to postoperative thoracic hemorrhage.

All patients underwent lobectomy or sublobar resection accompanied by mediastinal lymph node dissection, as necessary. Sputum suctioning was conducted for all 12 patients on postoperative days 1–3. Specifically, two patients received tracheal suctioning on three consecutive occasions, two underwent the procedure twice, and the remaining eight patients required suctioning only once post-surgery. Furthermore, the patients received general respiratory treatments as part of their routine care.

Within the framework of bronchoscopy-assisted postoperative respiratory management, all 12 patients successfully recovered without the need for postoperative reintubation. Following suction, notable reductions in inflammatory markers (including white blood cell counts, plasma C-reactive protein, and procalcitonin levels) were observed. No mortality occurred during hospitalization. The average postoperative hospital stay duration was 8.0 ± 5.5 days. Notably, one patient experienced an extended hospital stay of 25 days owing to postoperative paralytic ileus and hypoalbuminemia relapse. Three patients encountered minor complications, including atrial fibrillation.

Among the patients assessed as not requiring bronchoscopy-assisted suctioning, 71 experienced delayed discharge (postoperative hospitalization > 5 days) owing to various pulmonary complications such as atelectasis (*n* = 8), pneumonia (*n* = 21), prolonged air leak (>7 days) (*n* = 31), and pleural effusion (*n* = 10) (Fig. 1). Only one patient required reintubation owing to acute heart failure. Importantly, no reintubation was performed owing to pulmonary complications.

All patients underwent a follow-up phone call 1 week after discharge; no significant events necessitating emergency intervention were reported. Subsequently, three of the 12 patients exhibited evidence of pulmonary exudate or atelectasis on chest CT scans during the 3-week post-discharge follow-up. Importantly, these patients did not require readmission to the hospital for specialized treatment; instead, they received prescribed medications and scheduled follow-up care.

## Discussion

Postoperative respiratory complications remain a significant concern for patients with lung cancer, particularly those with compromised respiratory function and impaired cough reflexes due to neurological or muscular disorders. Managing retained airway secretions in this population is challenging, as ineffective secretion clearance can lead to complications such as atelectasis, pneumonia, and acute respiratory distress syndrome. Our study investigated the use of endotracheal suctioning via bronchoscopy as a potential strategy to address these issues. While this approach effectively facilitated secretion clearance and improved airway patency, several key considerations must be taken into account, including the optimal timing of intervention, the potential risks of procedure-induced airway inflammation, and the need for standardized assessment tools to guide clinical decision-making. A thorough evaluation of these factors is essential to define the role of bronchoscopy-assisted respiratory management in improving postoperative outcomes for high-risk lung cancer patients.

Weak cough strength and moderate-to-abundant secretions are strong predictors of unsuccessful extubation in critically ill patients following a spontaneous breathing trial^[6]^. Patients with weak cough (grades 0–2) are four times more likely to have unsuccessful extubations, whereas those with moderate-to-abundant secretions are more than eight times as likely. Our study successfully applied the airway competence assessment model to guide respiratory management in the immediate postoperative phase in patients with lung cancer. Specifically, we tailored our approach by reserving bronchoscopy-assisted postoperative respiratory management for those with weak cough (grades 0–2) and moderate or greater secretions, thus mitigating the need for reintubation even in individuals with neuromuscular disorders or advanced age. Our study underscores the potential of this assessment model to effectively guide airway management in high-risk patients. However, further studies with larger and more diverse populations are required to confirm their efficacy.

Efficient secretion removal is crucial for patient care; however, blind negative-pressure aspiration can damage the airway mucosa and leave secretions in place, worsening the patient’s condition^[16]^. In contrast, bronchoscopic secretion removal enables precise and thorough secretion removal while minimizing mucosal damage^[9]^. However, several factors must be considered when performing this procedure. First, early bronchoscopy intervention is crucial and should be optimally performed within 24–48 hours after surgery to prevent disease progression and minimize treatment complexity. We consistently perform this procedure in our practice within 2 days after extubation. Second, bronchoscopy must be gently performed by experienced endoscopists to minimize respiratory irritation and reduce the risk of adverse effects. Third, the duration of the bronchoscopy procedures should be minimized while pursuing thorough secretion removal.

Optimization of respiratory function before, during, and after surgery is crucial. General improvement measures include respiratory physiotherapy, which involves educating and supervising patients to ensure effective sputum clearance, enhancing inspiratory muscle strength, and practicing deep breathing exercises.^[17-19]^ Additionally, drug therapies such as inhaled β-agonists, inhaled steroids, inhaled mucolytics, and prophylactic postoperative antibiotics can improve pulmonary function to prevent respiratory infections^[20]^.

Our study has notable limitations. First, it relied on the subjective judgment of parameters that currently lack validated measures for critically ill patients. However, the use of a single observer was intended to ensure consistency when applying the classification system. Second, our analysis was limited to only two categories, potentially biasing the results toward null findings owing to random misclassification. Misclassification, such as patients with weak cough strength being incorrectly categorized as moderate-to-strong and vice versa may have led to the overuse of bronchoscopic suctioning in these groups. Consequently, future studies that incorporate more objective and validated measures and include multiple observers are crucial to generalize and refine these findings. Third, our sample size was relatively small, reflecting the rarity and highly selective nature of lung cancer patients with impaired cough strength and moderate-to-abundant secretions. Future studies with larger, more diverse cohorts are needed to validate these results and strengthen the conclusions for broader clinical application.

In summary, our study highlighted the efficacy of bronchoscopy-assisted postoperative respiratory management as a successful strategy to mitigate postoperative respiratory complications in patients with lung cancer and impaired cough strength. Our findings challenge the notion that weak airway competence is a contraindication for lung surgery. Instead, the airway competence assessment model has emerged as a valuable tool to guide bronchoscopy-assisted respiratory management for postoperative patients with lung cancer. These results underscore the significance of early bronchoscopic intervention and meticulous evaluation of airway secretions and cough strength, collectively contributing to the optimization of patient outcomes.

## Data Availability

The datasets analyzed during the current study are available from the corresponding author upon reasonable request.

## Research registration unique identifying number (UIN)

This study is a retrospective cohort study and was not prospectively registered in a public database. As the study did not involve prospective recruitment of participants, registration was not required. Therefore, no Unique Identifying Number (UIN) is applicable.
